# Increasing confidence and competence in supporting behaviour change in physiotherapy practice using Making Every Contact Count Healthy Conversation Skills: a before and after evaluation

**DOI:** 10.1186/s12913-025-12513-2

**Published:** 2025-06-04

**Authors:** Amelia Parchment, Wendy Lawrence, Em Rahman, Nick Townsend, Elaine Wainwright, David Wainwright

**Affiliations:** 1 Department for Health, University of Bath, Bath, BA2 7AY England, UK; 2 Aberdeen Centre for Arthritis and Musculoskeletal Health (Epidemiology Group), School of Medicine, Medical Sciences and Nutrition, University of Aberdeen, Aberdeen, AB25 2ZD UK; 3 Primary Care, Population Science and Medical Education, Faculty of Medicine, University of Southampton, Southampton, SO16 6YD England, UK; 4 Centre for Exercise, Nutrition and Health Sciences, School for Policy Studies, University of Bristol, Bristol, BS8 1TZ England, UK; 5 Public Health Workforce Development, NHS England- South East, Southampton, SO16 0AS England, UK; 6 NIHR Applied Research Collaboration for Greater Manchester, University of Manchester, Manchester, M13 9PL UK

**Keywords:** Brief intervention, Physiotherapy, Behaviour change, Making every contact count, Healthy conversation skills

## Abstract

**Aim:**

To a) evaluate the impact of Making Every Contact Count Healthy Conversation Skills (MECC HCS) training on the confidence and competence of physiotherapists in supporting patient behaviour change, and b) evaluate perceived acceptability, barriers and facilitators to implementing MECC HCS, following training.

**Methods:**

A before and after evaluation design was employed. MECC HCS training took place in October and December 2021. A range of measures were taken directly before training, directly after training, at 6- to 12- week follow-up and at 6- month follow-up. These measures related to confidence in delivering MECC HCS and supporting behaviour change in patients, competence in doing so, and perceived acceptability of utilising MECC HCS as a brief intervention to support behaviour change in practice.

**Results:**

MECC HCS training had significant positive impacts on the confidence and competence of physiotherapists in using MECC HCS skills to support patient behaviour change. Physiotherapists found training highly valuable and felt that implementing MECC HCS was acceptable within their practice. ‘Intentions’ and ‘Social/ Professional Role and Identity’ were key enablers to MECC HCS implementation at 6 months post- training.

**Conclusions:**

MECC HCS training may contribute to closing the gap between evidence-based recommendations and the practice of physiotherapists in relation to health promotion and supporting patient behaviour change and self-management.

**Supplementary Information:**

The online version contains supplementary material available at 10.1186/s12913-025-12513-2.

## Introduction

Roughly one third of the UK population live with a musculoskeletal (MSK) condition [73]. Many of these conditions cause persistent or chronic pain, lasting more than three months, and are associated with lower perceived quality of life, higher likelihood of depression, and reduced employment [24, 29, 68]. The clear and growing impact of MSK conditions and chronic pain on individuals, their employers, health services, and the wider economy, has prompted the development of long-term, strategic NHS plans, focusing on prevention and self-management of these conditions in clinical settings [56, 61].

Emphasis on prevention aims to reduce the risk of developing long-term health conditions (primary prevention) or lessen the impact of those that already exist (secondary prevention). Significant risk factors for onset and exacerbation of MSK conditions and chronic pain, such as physical inactivity, obesity, and stress, are therefore recommended targets for brief intervention and health promotion [16]. Supporting self-management can empower patients to become independent, active managers of their health condition and move away from a passive treatment approach that is focussed on cure [31]. Previous research shows that engaging in active self-management strategies for MSK conditions, such as physical activity, is associated with reduced use of healthcare services and lower levels of pain-related disability [10]. Similarly, active self-management interventions for chronic pain have been found to improve pain intensity, quality of life, and mental health [47].

Despite these promising findings, some people living with MSK conditions and pain do not feel confident to self-manage [46] and need person-centred support from healthcare professionals in taking first steps towards active self-management and improving lifestyle behaviours [22, 35, 69]. Person-centred support has been regarded highly important by patients with MSK conditions [15, 38], and focuses on the context, goals, values, knowledge and needs of an individual, in a collaborative, shared decision-making process between themselves and their healthcare professional [19].

Physiotherapists work with people presenting with MSK conditions and pain more than any other patient group [14], and are therefore uniquely placed to promote prevention and support self-management with these individuals [39]. However, key opportunities for this are frequently missed during clinical interactions [21, 58]. International evidence shows that physiotherapists experience difficulties integrating person-centred principles into routine practice [18] and don’t always feel they have the skills to promote health [74] or support self-management [32, 72]. Some report most frequently giving advice or information to facilitate behaviour change to enhance patient self-management [32]. However, this alone is usually insufficient to change behaviour, as individuals must also feel able and motivated to change [37, 48]. Moreover, focus is often placed on addressing biomechanical factors to increase likelihood of short-term recovery, instead of adopting a holistic approach to supporting long-term, health-promoting behaviour change [32, 45, 72]. Although many physiotherapists believe it is important and part of their role to promote long-term health, disease prevention and self-management in patients with MSK conditions and pain [32, 72, 74], there is a clear need for training opportunities to develop their confidence and competence in doing so [26, 40, 70].

**Fig. 1:**
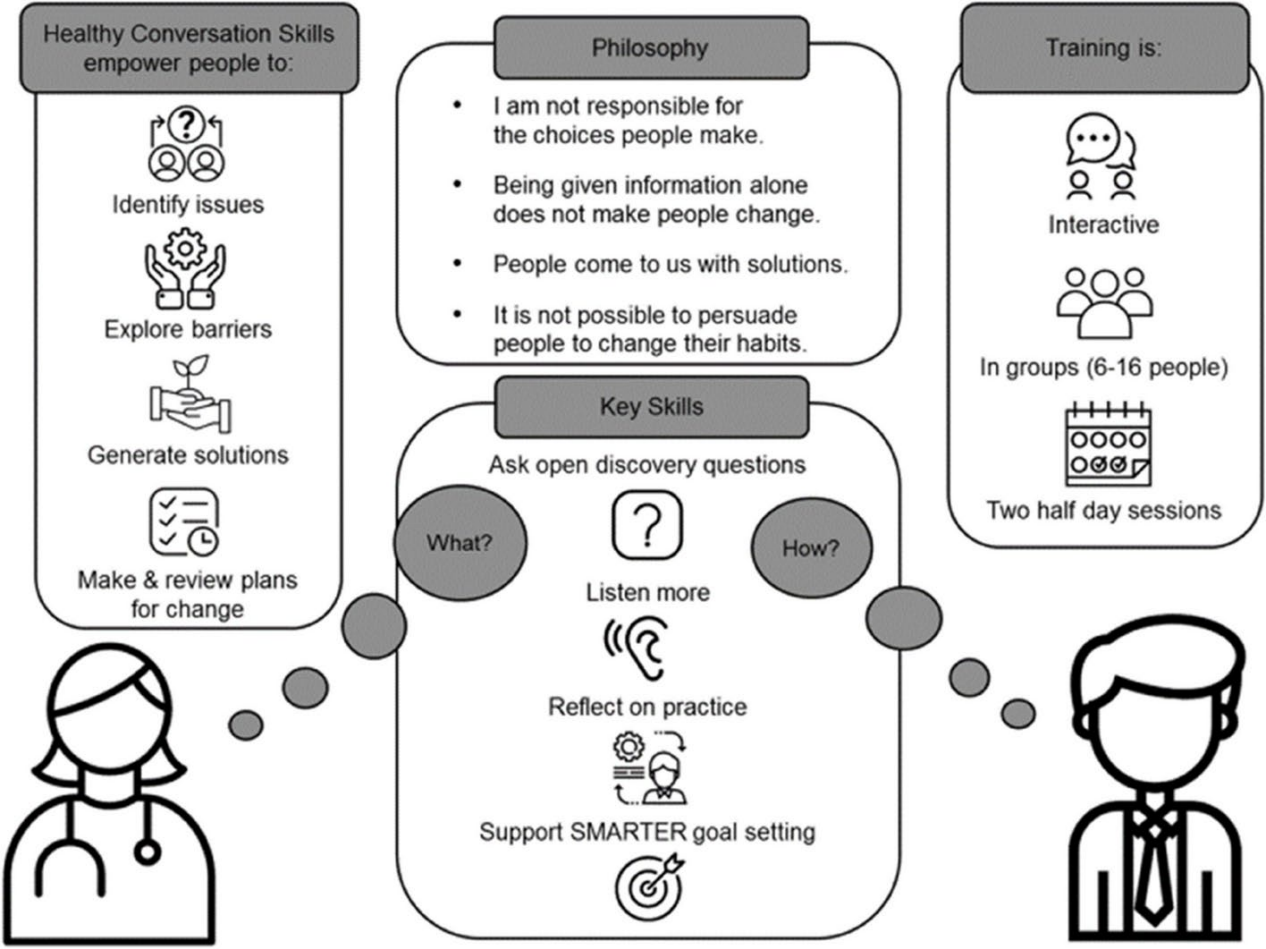
Healthy Conversation Skills philosophy, skills and training delivery (adapted from [28]). *Note*. SMARTER: Specific, Measurable, Action-oriented, Realistic, Timed, Evaluated, Reviewed

Healthy Conversation Skills (HCS) is an approach to behaviour change that empowers healthcare professionals to communicate within their practice in a person-centred way. Skills are developed for exploring the world and context of patients and supporting patients to find their own solutions and plan for change (Fig. 1) [8, 44]. Underpinned by the Taxonomy of Behaviour Change Techniques (BCTs) [52] and based on Social Cognitive Theory [4, 5], BCTs such as problem solving and goal setting are employed by training facilitators to build the self-efficacy of healthcare professionals in supporting patient health behaviour change. Healthcare professionals then help to build self-efficacy in their patients by employing the same BCTs during routine practice. Self-efficacy is one’s belief in their ability or competence to undertake a particular action [5]. Higher self-efficacy is associated with more healthful behaviours [5, 6] and increased physical functioning, engagement in physical activity and work status for those with MSK conditions and pain [49].

A range of evaluations have showed that engaging in HCS training increases practitioner confidence and competence in supporting behaviour change [9, 17, 42]. Patients who engage with HCS-trained practitioners have also been found to set more behaviour change goals, make more positive changes to health behaviours and experience higher satisfaction with their care than controls [1, 43, 60]. Recently, HCS has been integrated into the Wessex model of ‘Making Every Contact Count’ (MECC); a government initiative to embed prevention and health promotion into everyday healthcare practice [55]. Physiotherapists have been identified as potential key drivers for this initiative within MSK and pain settings and are expected to utilise all opportunities to ‘MECC’ with patients in routine consultations [61]. Physiotherapists already trained in the Wessex model of MECC HCS find the intervention highly acceptable, appropriate and feasible within their role [58]. Moreover, qualitative findings show that they believe the approach a) facilitates a holistic, person-centred approach to care, b) promotes long-term self-management and prevention, and c) improves their practice. Further organisational support is, however, needed to sustain MECC HCS within physiotherapy services and facilitate cultural change [59].

No research has yet explored the pre- to post-, and longer-term impact of MECC HCS training on the perceptions, self-efficacy and practice of physiotherapists in relation to supporting behaviour change. Positive findings may encourage further roll out of MECC HCS training in MSK and pain services, equipping physiotherapists with the skills and confidence to support behaviour change in an empowering, person-centred way.

The primary aims of the current study were to therefore a) evaluate the impact of MECC HCS training on physiotherapists’ perceived confidence in supporting behaviour change in patients with MSK conditions and pain and perceived usefulness and importance of supporting behaviour change in practice and; b) evaluate the impact of MECC HCS training on physiotherapists’ competence in using Healthy Conversation Skills to support behaviour change in patients with MSK conditions and pain. A secondary aim was to assess physiotherapists’ perceived acceptability of and barriers and facilitators to implementing MECC HCS in their practice, following training.

## Methods

### Ethics

This study was approved by the Health Research Authority (HRA) and the Research Ethics Committee (REC) (reference 21/EE/0107) in addition to University of Bath’s Research Ethics Approval Committee for Health (REACH) (reference EP 20/21 060).

### Design

A before and after evaluation design was employed to explore the impact of Making Every Contact Count (MECC) Healthy Conversation Skills (HCS) training on physiotherapists’ a) perceived usefulness, importance, and confidence in relation to supporting behaviour change in patients with MSK conditions and pain, and b) competence in using MECC HCS to support behaviour change in these patients. Measures were taken directly before (pre-) MECC HCS training, directly after (post-) training and at 6- to 12-week follow-up. Participants were then asked to complete measures at 6- month follow-up in order to explore long-term impact of MECC HCS training on supporting behaviour change in patients (compared to a ‘treatment as usual’ reference group), perceived acceptability of MECC HCS, and barriers and facilitators to implementation.

### Participants

With permission from local NHS Research and Development teams, physiotherapy leads within eight NHS trusts in Wessex, South-East and South-West regions of England were contacted by the lead researcher, via email, informing them of the research study and inviting their physiotherapy teams to take part. Participant information sheets were attached to these emails for dissemination within teams. Physiotherapists were eligible to participate in the study on the basis that they routinely worked with patients presenting with MSK conditions and pain and had received no formal MECC or HCS training prior to their participation. All physiotherapists that expressed interest in the study were directed to contact the lead researcher, who provided further detail on involvement, explained the format of training (2 X 3-h online sessions, delivered one or two weeks apart, during working hours), and shared a link to the informed consent form. Two options of training dates (October 2021 or December 2021) were given. After confirming their training date and signing the informed consent form, physiotherapists were directed, via online link, to complete the pre- training evaluation measure (detailed below). Those who expressed interest but a) were not available on either training date or b) contacted the lead researcher after December 2021, were invited to take part as ‘reference group’ physiotherapists, who would deliver treatment as usual (TAU) to patients and complete measures assessing competence in supporting behaviour change at 6- month follow-up. These participants were informed that they had the opportunity to receive formal MECC HCS training at a later date, after the study had finished.

### MECC HCS training and setting

MECC HCS training was delivered to two cohorts of physiotherapists in October and December 2021. Training was adapted for delivery in an online format, via Zoom, due to the COVID-19 pandemic and related government restrictions. Previous research has shown face-to-face HCS training has a positive impact on the confidence and competence of practitioners in supporting behaviour change [42, 44]. Since the pandemic, it has been developed and evaluated as a shorter, 90-min, virtually delivered training programme, successfully enhancing the skills of frontline workers in having supportive conversations with people impacted by COVID-19 [75]. This is the first study to evaluate the full MECC HCS programme, adapted for virtual delivery to those supporting behaviour change in the public. Physiotherapists were required to attend the full MECC HCS training programme, consisting of 2 X 3-h sessions delivered one to two weeks apart, for full accreditation by the Royal Society for Public Health to implement the brief intervention in practice. Aiming to build physiotherapists’ self-efficacy for having health behaviour related conversations with patients and support behaviour change, the trainers modelled the HCS philosophy and skills throughout training. For example, trainees were empowered to identify solutions and take first steps towards goals in the same way that they would empower their patients to do so, following training. Developing mastery experience through, for example, goal setting, is argued one of the most effective mechanism through which self-efficacy is built [7]. MECC HCS training activities have been mapped to the Taxonomy of Behaviour Change Techniques [51] in order to demonstrate how training can enable behaviour change in trainees (Appendix 1). The programme employed an active, participatory and interactive approach to learning, with the use of breakout rooms and group work. One MECC HCS ‘super’-trainer and one co-trainer facilitated all sessions. A visual depiction of the MECC HCS training programme is presented in Fig. 1.

### Procedure and materials

#### Pre- and post- training evaluation

Physiotherapists were asked to complete a pre- training evaluation form directly before the first online training session, and a post- training evaluation form at the end of the second and final training session (Appendix 2). Both were accessed online via JISC Online Surveys. These evaluation forms contained three questions examining changes in 1) perceived usefulness, 2) importance and 3) confidence in relation to supporting behaviour change in patients. Level of confidence was used as a proxy for self-efficacy, as in other similar studies [9, 25]. All questions were based around a 10-point Likert-type scale that ran from 1 to 10 and have been described in more detail elsewhere [9], and used previously to evaluate MECC HCS [44, 75].

Physiotherapists were also provided with four written statements relating to health and wellbeing issues, made by hypothetical patients, and were asked to type how they would verbally respond to these statements in their usual practice. These four written statements were developed and piloted previously for the evaluation of HCS and have been used for measuring staff HCS competence in a range studies [9, 42, 44]. A previously developed coding matrix [42] was used to score responses into one of eight possible categories (see Appendix 3, based on the extent to which they supported the patient to reflect on their problem and identify their own solutions (facilitating an empowering, person-centred approach to supporting behaviour change). A competence score of 1, for example, was given when physiotherapists responded by giving information by telling or making suggestions, and a competence score of 7 was given when they used open discovery questions (ODQs; Skill 1) to explore the context of the patient and support them to reflect and plan for change. Competence was independently assessed by two researchers to ensure consistency, and discrepancies were reviewed and discussed until agreement was reached.

The post- training evaluation form additionally asked physiotherapists to rate how valuable they felt the training was for their practice on a 5-point Likert-type scale, from 1 (not at all valuable) to 5 (extremely valuable).

#### 6- to 12- week follow-up (follow-up 1)

All physiotherapists that had completed training were emailed by the lead researcher, one-week post- training, to arrange follow up phone calls in order to assess medium-term impact of MECC HCS on practice. These calls were made between 6 to 12 weeks after completion of training to allow time for skills to be practised and implemented with patients in routine physiotherapy care. Variation in length of time between training and 6- to 12- week follow-up phone calls was due to capacity limitations, annual leave and sickness absence of the participants.

Consent to audio-record for transcribing purposes was obtained at the beginning of the phone call. During the phone calls, physiotherapists were first asked to reflect on their use of MECC HCS in practice with patients and share experiences of supporting behaviour change since attending training, in order to assess competence in the four HCS. Using a semi-structured discussion guide (Appendix 4), the lead researcher prompted the physiotherapist to think of how a health and/or wellbeing-related conversation with a patient had started; how it had progressed and how the physiotherapist responded (Skill 1: use of ODQs); who did the majority of talking/listening in the conversation (Skill 2: listening instead of giving information/ making suggestions); how they helped to support patients to plan for change using Specific, Measurable, Action-oriented, Realistic, Timed, Evaluated, Reviewed (SMARTER) goals (Skill 4: SMARTER goal-setting) and; what went well during the conversation, how they used the skills developed during training, how their practice had changed, and what they could do differently in future (Skill 3: Reflecting on practice). This discussion guide was previously developed and piloted, and has been employed in other HCS evaluations [9, 28]. Audio-recordings were transcribed verbatim, and competencies were assessed and scored using a previously developed coding rubric (discussed below).

#### 6- month follow-up (follow-up 2)

Six months post- training, all physiotherapists (including ‘reference group’ physiotherapists) were emailed by the lead researcher and asked to complete a follow-up evaluation form, via JISC Online Surveys.

They were asked to a) rate their perceived usefulness, importance, and confidence in relation to supporting behaviour change in patients and b) type their verbal responses to health and wellbeing- related statements made by hypothetical patients (as seen above in ‘pre- and post- training evaluation’). The purpose was to evaluate impact of MECC HCS training, six months later, and to compare data of those who had been trained and those who had not been trained in MECC HCS.

Those who had been trained in MECC HCS and were delivering it in practice were asked to fill in additional measures to assess perceived acceptability of the brief intervention, barriers and enablers to implementation. The Theoretical Framework of Acceptability (TFA) questionnaire, a brief, adaptable, theoretically informed tool for use in healthcare settings, was used to quantitively assess acceptability [65]. The TFA [64] is comprised of seven component constructs (affective attitude, burden, ethicality, intervention coherence, opportunity costs, perceived effectiveness, and self-efficacy) that can help identify issues with intervention acceptability that could be addressed in order to enhance future implementation. The questionnaire was developed to help researchers assess healthcare intervention acceptability in response to the UK Medical Research Council (MRC) best practice guidance for evaluating complex interventions [53, 67].

The Theoretical Domains Framework (TDF) [12] informed the development of questions to assess barriers and enablers to MECC HCS implementation. The TDF is an integrative framework consisting of 14 theoretical domains of potential cognitive, affective, social and environmental influences on behaviour, and can be used to understand health professional behaviour in relation to implementation of evidence-based recommendations [2], such as the UK’s MECC initiative. This can improve understanding of factors contributing to the success of implementation and those that could be targeted in order to optimise future rollout. Questions from a previously developed TDF survey [30] were adapted to encompass supporting behaviour change using MECC HCS, i.e., *‘Delivering MECC HCS to support behaviour change with patients is something I rarely forget’*. Nine domains were included in the analysis: 1) Skills; 2) Social/Professional Role and Identity; 3) Beliefs about Capabilities; 4) Beliefs about Consequences; 5) Intentions; 6) Memory, 7) Attention and Decision Processes; 8) Environmental Context and Resources; 9) Social Influences; and Emotion. Three domains were not included in the questionnaire of the follow-up evaluation (Reinforcement, Goals, and Behavioural Regulation) as items were not found to discriminately measure them in the Huijg et al. [30] study mentioned above. Two further domains were omitted from analysis (Skills and Optimism) as physiotherapist responses to items measuring these domains were not complete.

### Analysis

Descriptive statistical analyses were used to report demographic information. Perceived confidence, usefulness and importance in relation to supporting behaviour change in patients with MSK conditions were described using medians and interquartile ranges (IQRs). Response style competence scores were described in the same way. These measures were taken at pre- training, post- training and at 6- month follow-up.

A previously developed coding rubric (Appendix 4) [9, 42] was used to score physiotherapists’ competence in use of the four HCS (asking open discovery questions, listening instead of giving information/ making suggestions, reflecting on practice, and supporting SMARTER goal setting) at 6- to 12- week follow-up. Data were taken from transcripts of phone calls, whereby physiotherapists were asked to share their experiences of supporting behaviour change in their practice since attending MECC HCS training. Each HCS was scored from 0–4 by one researcher, where 0 demonstrated no competence and 4 demonstrated the highest competence. Seven randomly selected transcripts (50%) were then double coded by a second researcher, before coding was compared and discussed. One hundred percent agreement between HCS competency scores of both researchers was reached, supporting validity of findings. Competence scores for each HCS were described using medians and IQRs. Competence scores for each participant were described by totalling HCS scores and dividing by four, to give an overall mean.

Changes in perceived confidence, usefulness and importance scores, pre- to post- training and at 6- month follow-up, were explored using Wilcoxon matched-pairs signed-rank tests, as were changes in response style competence scores. Mann–Whitney U tests were employed to examine any differences in competence scores at follow-up between physiotherapists who received MECC HCS training and were delivering the brief intervention in practice, and those who did not receive training and were continuing to give treatment as usual in practice.

Single perceived acceptability scores were calculated by computing physiotherapists’ total mean score on the seven Theoretical Framework of Acceptability components (1 = low acceptability to 5 = high acceptability). Items assessing ‘burden’ and ‘opportunity costs’ were reverse scored to ensure that higher scores always indicated higher acceptability. Scores for each TFA component were described using medians and IQRs.

Seven-point Likert scales (1 = strongly disagree to 7 = strongly agree) were used to explore barriers and enablers to MECC HCS implementation using questions informed by the Theoretical Domains Framework [12]. Items that were negatively worded were reverse scored. As in other similar studies [23, 27], mean values for each TDF domain were calculated by summing the scores for all items within the domain and dividing by the number of items. A lower mean value (< 6) indicated that the domain could pose as a barrier to delivering MECC HCS in physiotherapy practice, whilst a higher mean score (> 6) suggested that it was a potential enabler. Pairwise correlation coefficients were used to explore any relationships between TDF domains. Strength of association was described as very high (0.90–1.00), high (0.70–0.90), moderate (0.50–0.70), low (0.30–0.50 and negligible (0.00–0.30), with corresponding negative correlations [54]. Correlations that were moderate and higher are reported in this article.

Data were analysed using IMB SPSS Statistics 27.

## Results

A total of 43 physiotherapists working with people with MSK conditions and pain within UK NHS organisations were recruited and consented to participate in this study. Thirty-one received Making Every Contact Count Healthy Conversation Skills training in October or December 2021. The remaining twelve (‘reference group’ physiotherapists) did not receive training, continued giving treatment as usual (TAU) to their patients, and were contacted at 6- month follow-up. Of those that did receive training, 23 completed both pre- and post- training measures immediately before and after training (retention rate 74.2%), 14 participated in 6- to 12- week follow-up phone calls, and 11 completed measures at 6- month follow-up. The total retention rate for all participants, from pre- training to 6- month follow-up was 53.5%. The main reasons for drop-out were redeployment or capacity limitations due to the COVID-19 pandemic. Participant characteristics are shown in Table 1.

**Table 1:** Participant characteristics (*n* = 35)

**Variable**	**N**	**%**
**Intervention group physiotherapists**	**23**	
**Gender**		
Women	16	69.6
**Age**		
18–24	2	8.7
25–34	6	26.1
35–44	8	34.8
45–54	2	8.7
56–64	5	21.7
**Reference group physiotherapists**	**12**	
**Gender**		
Women	7	58.3
**Age**		
18–24	3	25.0
25–34	3	25.0
35–44	4	33.3
45–54	2	16.7

### Confidence in supporting behaviour change in patients with MSK conditions

Self-reported confidence of physiotherapists in supporting behaviour change in patients significantly increased, pre- (median (IQR) = 6 (5–8)) to post- (median (IQR) = 8 (7–8)) MECC HCS training (Z = −2.742, *p* = 0.006) (*n* = 23).

A separate analysis of data of those who also completed six- month follow up measures (*n* = 11) showed that their confidence significantly increased from pre- (median (IQR) = 6 (5–7)) to post-(median (IQR) = 8 (6–8)) training (Z = −2.558, *p* = 0.011), and this remained significant from pre- training to six- month follow up (median (IQR) = 8 (7–8)), (Z = −2.714, *p* = 0.007).

### Importance of supporting behaviour change in patients with MSK conditions

MECC HCS training did not, however, elicit a statistically significant change in the perceived importance of supporting behaviour change pre- post (Z = −0.643, *p* = 0.520) training (*n* = 23). Physiotherapists already scored high on this measure, pre- training (median (IQR) = 9 (8–10)), and this remained high at post- training (median (IQR) = 9 (8–10)).

These findings were similar when analysing the data of only those who additionally completed six- month follow up measures (*n* = 11). Their perceived importance of supporting behaviour change at pre- training was very high (median (IQR) = 10 (8–10)) and this remained very high at six- month follow up (median (IQR) = 10 (9–10)) (Z = −0.962, *p* = 0.336).

### Perceived usefulness of conversations with patients for supporting behaviour change

Self-reported usefulness of conversations for supporting behaviour change in patients significantly increased, pre- (median (IQR) = 7 (6–8)) to post- (median (IQR) = 8 (7–9)) MECC HCS training (Z = −2.069, *p* = 0.039) (*n* = 23).

For those who additionally completed six- month follow up measures (*n* = 11), an increase in perceived usefulness was also observed pre- training (median (IQR) = 6 (3–10)) to follow-up (median (IQR) = 8 (7–9)), but this change was not significant (Z = −1.488, *p* = 0.137). Further descriptive analyses did, however, show that at pre- training 27.3% of respondents scored 3 or below on self-reported usefulness of conversations (low perceived usefulness), and only 45.5% scored 7 or above (high perceived usefulness). In contrast, at six- month follow-up, 0% scored 3 or below and 81.9% scored 7 or above.

### Physiotherapist competence immediately post- training

**Fig. 2:**
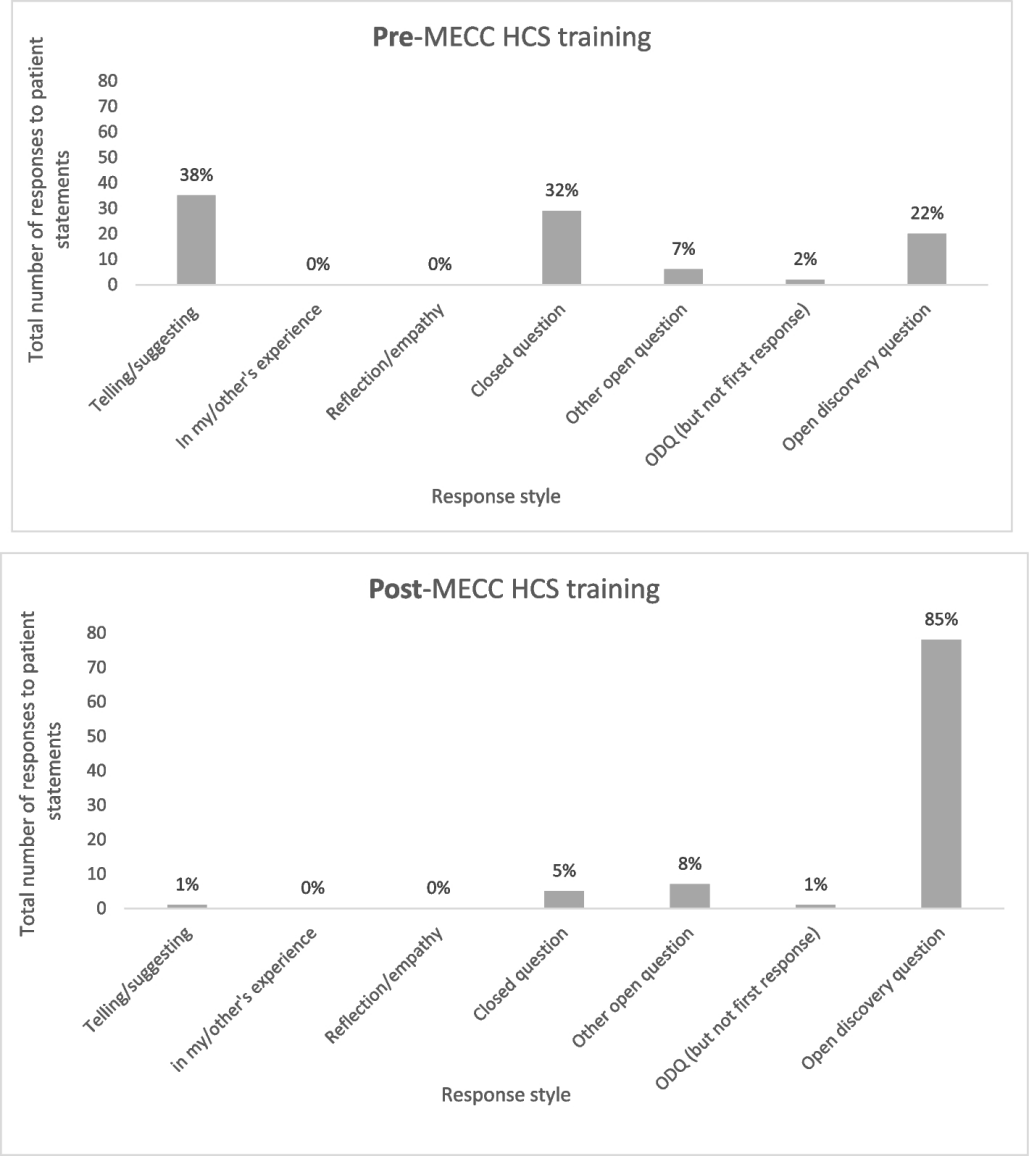
Change in physiotherapists’ response styles to patient health behaviour statements, pre- to post- Making Every Contact Count Healthy Conversation Skills (MECC HCS) training (n participants = 23; n responses = 92)

Physiotherapists responded to four patient statements regarding health and wellbeing significantly differently after training (*p* < 0.001) (Fig. 2). The number of open discovery questions physiotherapists responded with increased after training from 20 (22% of total (92) responses) to 78 (85% of total (92) responses), whilst responding with giving information/ making suggestions and using closed questions decreased from 35 to 1 and 29 to 5, respectively. This indicates that the physiotherapists had adopted a more person-centred, empowering approach to supporting behaviour change and their competence in using ODQs (Skill 1) had increased. One hundred percent (*n* = 23) of participants used at least one open discovery question after training, and 91% (*n* = 21) were using 3–4.

### Physiotherapist competence at 6- to 12- week follow-up

**Fig. 3:**
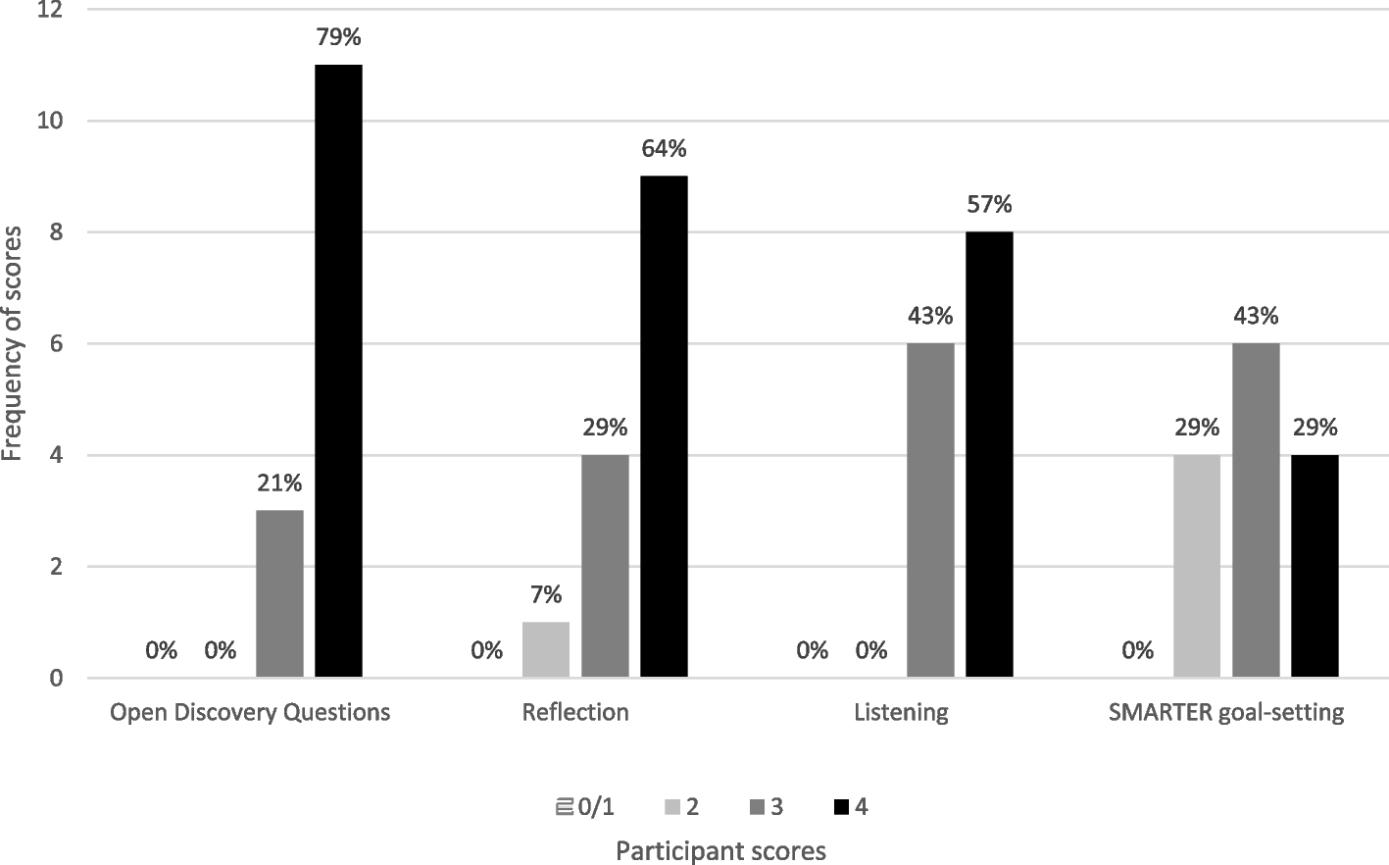
Physiotherapists’ scores on the four MECC HCS competencies, 6 to 12 weeks post- training (data extracted from follow-up calls; *n* = 14). *Note.* Scale 0–4: 0 = no competency; 1 = some demonstration of competency; 2 = moderate demonstration of competency; 3 = good demonstration of competency; 4 = strong demonstration of competency

Data of fourteen physiotherapists were available at 6- to 12- week follow-up. Competence scores for each skill (maximum score of 4) were plotted and median scores calculated. Variation in median scores, as shown in Fig. 3, suggests they were more competent in some Healthy Conversation Skills than others. For example, high levels of competence was demonstrated in asking open discovery questions (median (IQR) = 4 (3–4)), reflecting on practice (median (IQR) = 4 (2–4)), and listening rather than giving information (median (IQR) = 4 (3–4)), but physiotherapists did not always report supporting behaviour change in patients using SMARTER goal-setting (median (IQR) = 3 (2–4)). The mean overall competence rating for all intervention physiotherapists was 3.5 (SD: 0.36) (maximum score of 4; strong demonstration of competency).

### Physiotherapist competence at 6- month follow-up

**Fig. 4:**
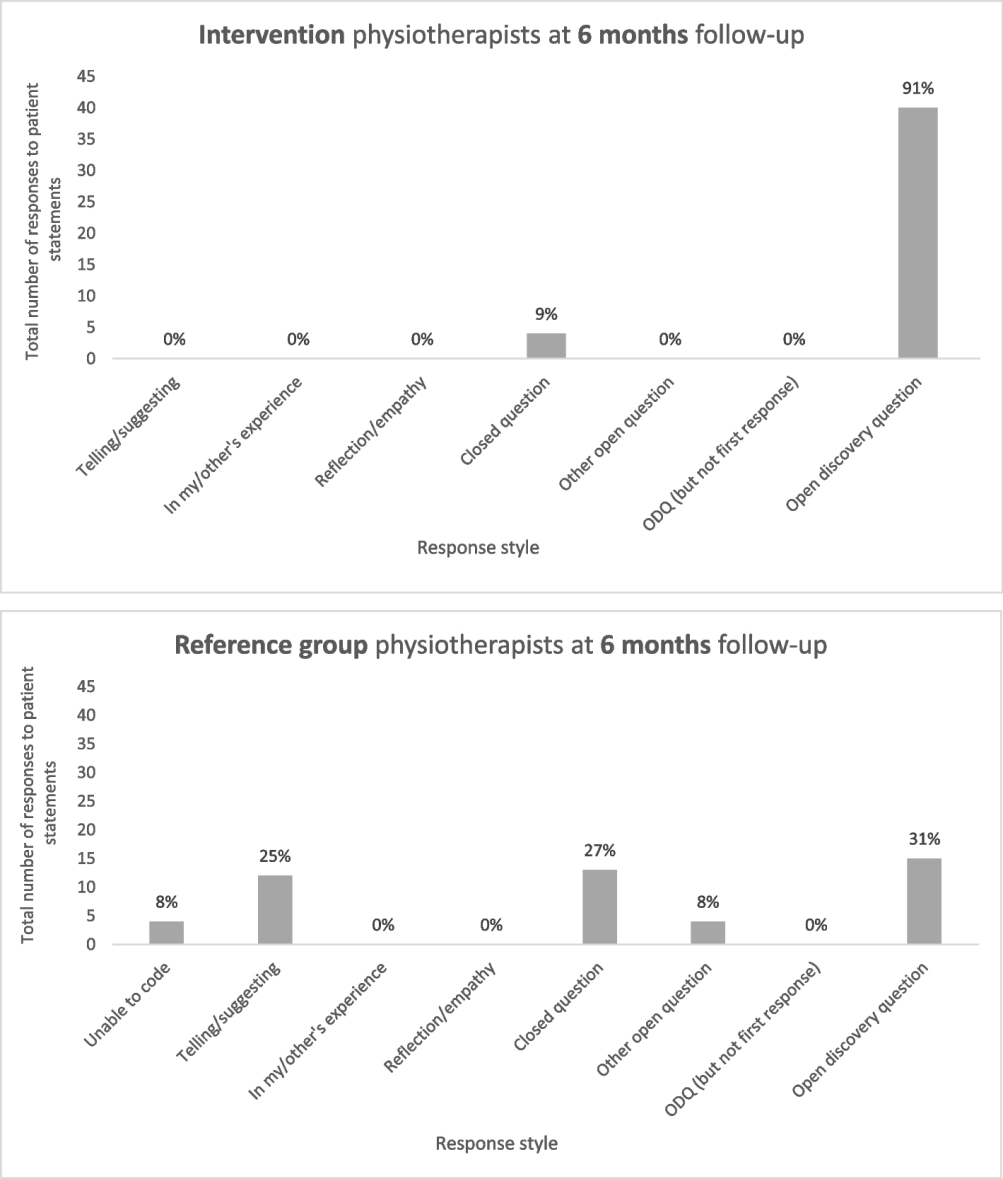
Response styles of physiotherapists trained in MECC HCS (*n* participants = 11,* n* responses = 44) vs. physiotherapists delivering treatment as usual (*n* participants = 12, *n* responses = 48), at 6- month follow-up

At 6- month follow-up, there were significant differences in the responses to patient health behaviour statements between physiotherapists trained in MECC HCS and those who had not received training (U = 402, *p* < 0.001) (Fig. 4). At this time point, 91% of responses of MECC HCS trained physiotherapists were open discovery questions, compared to 31% of responses of those who had not been trained. Reference group physiotherapists responded with mostly telling/suggesting and closed questions. Those trained in MECC HCS were thus more competent in using ODQs to explore the context of the patient and support them to reflect and plan for change (suggesting a person-centred, empowering approach) than those who had not been trained.

### Perceived acceptability of MECC HCS and barriers and facilitators to implementation

On a scale of 1 to 5 (1 = not at all valuable, 5 = extremely valuable), 78% of physiotherapists (18/23) rated the MECC HCS training a 4 or 5, thus, valuable or extremely valuable. The remaining 22% felt that the training had at least some value for physiotherapy practice.

Perceived acceptability of implementing MECC HCS as a brief intervention in physiotherapy practice was measured using the Theoretical Framework of Acceptability questionnaire [65]. The mean score for physiotherapists at 6- month follow-up (*n* = 11) was 3.9 (/5, acceptable). Median scores for all TFA constructs suggest that physiotherapists; i) liked MECC HCS as an intervention (affective attitude) (median (IQR) = 4 (2–5)); ii) felt that it took a little effort to engage with (burden) (median (IQR) = 4 (2–4)); iii) felt that it was fair for use with patients with MSK conditions (ethicality) (median (IQR) = 4 (3–5)); iv) agreed that it improved their ability to support behaviour change with patients (perceived effectiveness) (median (IQR) = 4 (4–5)); v) agreed that it is clear how it could improve health and wellbeing- related conversations with patients (intervention coherence) (median (IQR) = 4 (4–5)); vi) felt they were confident in delivering MECC HCS to patients (self-efficacy) (median (IQR) = 4 (3–4)); and vii) disagreed that it interfered with other priorities (opportunity costs) (median (IQR) = 4 (2–5)).

Analysis of responses to TDF items at 6- month follow-up showed that two domains (‘Social/ Professional Role and Identity’, and ‘Intentions’) scored > 6, suggesting they were potential enablers for implementing MECC HCS in practice. ‘Skills’ and ‘Beliefs about Consequences’ were just below this threshold. ‘Environmental Context and Resources’ and ‘Memory, Attention and Decision Processes’ had the lowest mean score, suggesting these were the biggest perceived barriers to MECC HCS implementation (see Table 2).

**Table 2:** Mean scores for barriers and enablers according to TDF survey [30] as reported by physiotherapists at 6- month follow-up (*n* = 11)

**TDF domain**	**Definition**	**Mean (SD)**
Skills	An ability or proficiency acquired through practice	5.71 (0.72)
Social /Professional Role and Identity	A coherent set of behaviours and displayed personal qualities of an individual in a social or work setting	6.24 (1.02)
Beliefs about Capabilities	Acceptance of the truth, reality or validity about an ability, talent, or facility that a person can put to constructive use	5.06 (1.11)
Beliefs about Consequences	Acceptance of the truth, reality or validity about outcomes of a behaviour in a given situation	5.87 (0.85)
Intentions	A conscious decision to perform a behaviour or a resolve to act in a certain way	6.60 (0.65)
Environmental Context and Resources	Any circumstance of a person’s situation or environment that discourages or encourages the development of skills and abilities, independence, social competence, and adaptive behaviour	4.49 (1.16)
Memory. Attention and Decision Processes	The ability to retain information, focus selectively on aspects of the environment, and choose between two or more alternatives	4.58 (1.10)
Social Influences	Those interpersonal processes that can cause individuals to change their thoughts, feelings, or behaviours	5.23 (0.68)
Emotion	A complex reaction pattern, involving experiential, behavioural, and physiological elements, by which the individual attempts to deal with a personally significant matter or event	4.86 (1.14)

Strong positive correlations were observed between a) ‘Beliefs about Capabilities’ and ‘Beliefs about Consequences’ (0.75, *p* = 0.008), and b) ‘Social Influences’ and ‘Intentions’ (0.81, *p* = 0.002). Moderate positive correlations were found between a) ‘Beliefs about Capabilities’ and ‘Intentions’ (0.69, *p* = 0.019), and b) ‘Memory, Attention and Decision Processes’ and ‘Emotion’.

## Discussion

This is the first known study to evaluate MECC HCS within physiotherapy services from pre- to post- training, and at longer-term follow up. The training programme had significant positive impacts on the confidence and competence of physiotherapists in supporting behaviour change in patients. Physiotherapists found training highly valuable and felt that implementing MECC HCS was acceptable in their practice. ‘Intentions’ and ‘Social/ Professional Role and Identity’ were identified using the TDF survey as key enablers to MECC HCS implementation at 6 months post- training. This is the first time the full MECC HCS programme has been evaluated in its adapted online format. It appears that it could be just as successful in increasing the competence and confidence of healthcare professionals in supporting behaviour change as in its face-to-face format [17, 44]. Findings are discussed below as they answer each study aim.

### What is the impact of MECC HCS training on physiotherapists’ perceived usefulness, importance and confidence in relation to supporting behaviour change in patients with MSK conditions and pain?

Consistent with evaluations of HCS involving other staff groups [9, 17, 28, 44], MECC HCS training was successful in increasing the confidence/ self-efficacy of physiotherapists in supporting behaviour change in patients. This was still evident at 6- month follow-up, highlighting the potential long-term impact of training. This finding is particularly promising, since some physiotherapists report a lack of confidence as a barrier to engaging in health promotion or supporting behaviour change [57]. There are reported gaps between evidence-based guidance and the practice of physiotherapists in relation to health promotion and supporting self-management, internationally [21, 72]. MECC HCS training might be an important opportunity for increasing physiotherapists’ uptake of both. In the UK, this could be pivotal for meeting the goals of the NHS in relation to promoting prevention and self-management in patients with MSK conditions [61].

No significant changes in perceived importance of supporting behaviour change were found pre- to post- training, or at follow up. Perceived importance was instead found to be very high at pre- training, and this stayed constant at all time points. These findings align with those of other studies, showing that physiotherapists perceive health promotion and supporting behaviour change as important within their role [72, 74] and believe supporting self-management is integral for effective MSK patient care [32]. They report trying to facilitate patient behaviour change in order to enhance self-management [32, 72]. Physiotherapists in the present study are thus likely to have had similar perceptions regarding their own role in supporting health promotion and behaviour change, and the importance of this within their practice. In support of this, 'Social/ Professional Role and Identity’ as a TDF domain was found to be an enabler of MECC HCS implementation, as discussed later.

Perceived usefulness of conversations for supporting behaviour change increased significantly from pre- training to post- training, however, the observed increase from pre- training to 6- month follow-up did not stay significant. These findings suggest that physiotherapists felt that developed HCS were bringing additional value to conversations with patients, directly after training. Qualitative data that has been published elsewhere, as part of a wider project, shows that physiotherapists and patients with MSK conditions advocate for the use of ‘how’ and ‘what’ questions and listening instead of giving information/ advice for facilitating productive, person-centred care [59]. Moreover, SMARTER-goal setting has been considered highly valuable for supporting patients to plan for change [44]. Our observed increase in perceived usefulness aligns with the findings of other HCS evaluations [17, 28, 44] and suggests that engaging in MECC HCS training develops skills for having useful, productive, person-centred conversations with patients. Although perceived usefulness of conversations also increased from pre- training to 6- month follow-up, lack of statistical significance may have been due to participant attrition. Loss to follow-up will be discussed later as a limitation of this study.

### What is the impact of MECC HCS training on physiotherapists’ competence in using Healthy Conversation Skills to support behaviour change in patients with MSK conditions and pain?

Physiotherapists demonstrated significantly increased competence in using ‘open discovery’ questions to support behaviour change, directly after MECC HCS training. They were therefore adopting a more exploratory approach to responding to health and wellbeing-related statements by hypothetical patients; using ‘how’ and ‘what’ questions to explore context, help patients to identify their own solutions and plan for change. In contrast, responding to patient statements by giving information/ telling significantly decreased, post- training. These findings reflect a shift towards an empowering, person-centred approach to supporting behaviour change, and one which is relevant to the patient’s world and based on the patient’s own agenda, making change more likely [1, 43]. MECC HCS therefore had a positive impact on the short-term practice of physiotherapists in supporting behaviour change in patients with MSK conditions and pain.

At six- to twelve- week follow-up, physiotherapists continued to demonstrate high competence in using ‘open discovery’ questions and most of the other HCS. They were found, for example, to also be highly competent in listening instead of giving information/ making suggestions (Skill 2). By listening more during patient interactions, physiotherapists were again facilitating a person-centred approach to supporting behaviour change [15, 41]. Whilst information delivery alone is highly unlikely to change behaviour [11, 62, 71], listening is a skill that is valued by MSK patients in clinical settings [50] and advocated for enabling a fuller understanding of factors such as personal engagement and motivation for behaviour change [20]. Both are key drivers of change in those with chronic conditions [20]. One study focusing specifically on chronic pain showed that active listening helped physiotherapists to build rapport with patients. This allowed for assessment of patients' readiness to change, perceived importance of and confidence in their ability to change behaviours to enhance self-management; key associated factors within the Motivational Model of Pain Self-Management [34]. Increased understanding of these factors informed the physiotherapist’s subsequent use of BCTs, aimed at increasing patient self-efficacy and engagement in health behaviour change [26]. Listening may therefore be a highly important HCS for physiotherapists in the initial stages of supporting behaviour change, for understanding patient context and adapting behaviour change support to fit individual needs.

Physiotherapists were also highly competent in reflecting on practice (Skill 3) at six- to twelve- week follow-up. Critically reflecting on practice, through problem-solving, has been evidenced to increase HCS trainees’ confidence/ self-efficacy in reorientating their practice and implementing HCS routinely [9]. Physiotherapists in the present study successfully reflected on how their practice had changed since training, what they were doing well to support behaviour change in patients and what they could do differently. Such critical reflection may be important for the continued development of skills and their sustained use within routine practice [33].

Lower competence was, however, found for SMARTER goal setting (Skill 4) at 6- to 12- week follow-up. Other studies have found limited use of SMARTER goal setting to support behaviour change by HCS trained practitioners [17, 33, 42], and have suggested that further follow-up support is needed for increasing competence in this particular HCS [42]. Time has, however, been reported as a major barrier to successful MECC HCS implementation within physiotherapy practice [59]. Physiotherapists may therefore be capable of using SMARTER goal setting to support behaviour change but feel that they don’t have the opportunity to regularly do so within their limited consultation time frames. This was highlighted in follow-up, qualitative interviews and could also be reflected by the present Theoretical Domains Framework [12] findings, in which ‘Environmental Context and Resources’ was the main barrier to MECC HCS implementation.

At six- month follow-up, physiotherapists trained in MECC HCS demonstrated sustained changes in practice, highlighting a potential long-term impact of training. They were using significantly more open discovery questions to support behaviour change than ‘reference group’ physiotherapists, who responded to patient health statements with mostly telling/suggesting and closed questions. Similar HCS evaluations have evidenced trained practitioners implementing empowering, person-centred HCS significantly more than controls, at least 1-year post- training [3, 42]. These findings are promising for the future practice of MECC HCS-trained physiotherapists and the potential outcomes for patients with MSK conditions and pain, since most skills seem to become embedded and sustained in routine care.

### How acceptable is MECC HCS to physiotherapists supporting patients with MSK conditions and pain, and what are the barriers and facilitators to implementing MECC HCS in routine practice?

Importantly, MECC HCS training was very well received by physiotherapists, and they found the intervention acceptable within their practice. Assessing acceptability from the healthcare professional’s perspective is considered a fundamental part of evaluating complex healthcare interventions. Intervention acceptability can impact uptake, the extent to which the intervention is delivered as intended and its overall effectiveness [53, 66]. This study was the first to evaluate retrospective acceptability of MECC HCS using a theoretically informed questionnaire based on seven component constructs [64, 65]. The Theoretical Framework of Acceptability (TFA) suggests that acceptability is multifaceted [64]. Subsequent development of the TFA questionnaire therefore aimed to allowed for the identification of constructs of acceptability that could be enhanced through intervention refinement [65]. Promisingly, the findings of the present study suggest that physiotherapists found MECC HCS acceptable across all constructs. Further work has also shown that MECC HS is highly acceptable to patients with MSK conditions and pain [60], however, further work is needed to evaluate acceptability and effectiveness of this brief intervention for patients on a wider scale.

‘Environmental Context and Resources’ and ‘Memory, Attention and Decision Processes’ were found to be key barriers to successful implementation of MECC HCS. Research involving physiotherapists already trained in and delivering MECC HCS has highlighted perceived barriers to implementation mostly relating to ‘Environmental Context and Resources’ on the TDF, such as lack of time, lack of support for ‘MECC’ from leadership and limited access to training and resources [58]. This suggests that organisational, system-level influences must be addressed in order to enhance implementation. ‘Memory, Attention and Decision Processes’ as a second key barrier may reflect the challenges faced by physiotherapists in remembering to implement skills due to their high workload and deciding between competing clinical priorities during practice. These challenges have been evidenced in other research assessing physiotherapists’ barriers to health promotion [36, 63].

It seemed that physiotherapists in the present study did, however, have the internal motivation to deliver MECC HCS, reflected by ‘Social/Professional Role and Identity’ and ‘Intentions’ as key enablers [2]. As mentioned earlier, physiotherapists believe it is important and part of their role to promote health and support behaviour change [36, 72, 74]. ‘Social/ Professional Role and Identity’ as an observed long-term enabler to MECC HCS implementation supports this. It is interesting to note that in a separate HCS evaluation, ‘Intentions’ to have behaviour change conversations improved from pre- to post- HCS training, but this was not sustained at 6–10 week follow up [28]. This TDF domain was, however, an enabler at 6- month follow-up in the present study. This suggests that physiotherapists were using goals and planning (BCTs employed during training) in the long term for supporting behaviour change within their practice [13, 27]. Future research should seek to understand the significant associations found between TDF domains in relation to MECC HCS implementation. Our preliminary findings show, for example, there is a relationship between ‘Social Influences’ and ‘Intentions’, however, data from more participants are needed to reach sufficient power and establish the characteristics of this relationship.

### Limitations

Although this study has several strengths, some key limitations must be addressed. Firstly, the sample size was smaller than anticipated due to participant attrition. This research was carried out during the COVID-19 pandemic and several physiotherapists no longer had capacity to participate in the study at follow-up. Those that did participate in the study may have been favourably disposed toward the principles of MECC and/or promoting prevention and behaviour change in healthcare settings, therefore, a bias in the sample cannot be ruled out. Future, post-pandemic research should aim to evaluate MECC HCS for physiotherapists on a larger scale. Increasing sample size would ensure that the study is adequately powered for detecting differences within and between groups that have and have not received MECC HCS training.

Secondly, this evaluation relied on self- report and data collected via phone calls conducted by the lead researcher. A risk of social desirability was therefore present and may have influenced the physiotherapists to overstate or exaggerate their responses. A range of evaluation tools and multiple data collection time-points were, however, used in an attempt to address this potential bias. Although some evaluation tools were not validated against other instruments, they have been piloted extensively, used in many other HCS evaluations and are described as fit-for-purpose [9, 42, 44].

Thirdly, not all TDF domains were included in the 6- month follow-up survey. Although the employed TDF survey is a valid tool for exploring barriers and enablers to intervention implementation, at the time of this study, it was not able to discriminately measure ‘Reinforcement’, ‘Goals’ and ‘Behavioural Regulation’ as TDF domains [30]. Data relating to ‘Skills’ and ‘Optimism’ were additionally omitted from analysis due to incomplete responses on these items at follow up. It is possible that these TDF domains could have been additional barriers or enablers to physiotherapists implementing MECC HCS in practice.

Finally, a ‘treatment as usual’ group was used within this study design; however, measures were taken from these participants only at 6- month follow-up. This was due to time and resource constraints as a result of the COVID-19 pandemic. Future work should employ a more controlled study design to further explore relationships between engagement in MECC HCS training and changes in physiotherapists’ competence and confidence in supporting behaviour change.

## Conclusion

This study indicates that MECC HCS training has a positive impact on the confidence and competence of physiotherapists in supporting behaviour change in patients with MSK conditions and pain. This seems sustained for at least 6 months, showing a potential long-term impact of training. Although barriers to implementation exist and must be addressed, MECC HCS is considered acceptable to physiotherapists within their practice, making uptake more likely. MECC HCS training may therefore contribute to closing the gap between evidence-based recommendations and the practice of physiotherapists in relation to health promotion and supporting self-management. This should be studied on a larger scale, employing a more controlled study design.

## Supplementary Information

Supplementary Material 1.

## Data Availability

Materials and data set are available from the corresponding author upon request.
