# Supplementary material for: Increasing confidence and competence in supporting behaviour change in physiotherapy practice using Making Every Contact Count Healthy Conversation Skills: a before and after evaluation
Source: BMC Health Serv Res. 2025 Jun 4;25:797. doi: 10.1186/s12913-025-12513-2 (PMC12135257; doi:10.1186/s12913-025-12513-2)
Supplement: Supplementary file 1 — Supplementary Material 1. [file 12913_2025_12513_MOESM1_ESM.docx]

**Appendices**

**Appendix 1: Healthy Conversation Skills training activities mapped to Taxonomy of Behaviour Change Techniques (Michie et al, 2013)**

| **Activity** | **Group No.** | **BCT Group** | **BCT No.** | | **BCT** | **Activity component** |
| --- | --- | --- | --- | --- | --- | --- |
| Overall, throughout both sessions | 1 | Goals & planning | 1.2  1.6 | | Problem-solving  Discrepancy between current behaviour & goal | How to use skills in practice – prompting trainee to generate/select strategies to overcome barriers & increase facilitators to HCS use, including “relapse prevention” & “coping planning” to avoid returning to any previous style of interacting.  Reflection on changing current practice – drawing trainee’s attention to discrepancies between current practice and plans/goals to incorporate HCS into practice. |
|  | 3 | Social support | 3.2  3.3 | | Social support (practical)  Social support (emotional) | Group training & pair work provides practical support (listening & sharing tips) for practising HCS in the training & later in the workplace.  Group training & pair work provides emotional support (encouragement/praise) for practising HCS. |
|  | 4 | Shaping knowledge | 4.1  4.2 | | Instruction on how to perform the behaviour  Information on antecedents | Skills training, including advice & agreement for how to develop questions, support SMARTER planning etc.  Review what predicts behaviour, e.g., when running out of time can revert to telling or suggesting. |
|  | 6 | Comparison of behaviour | 6.1 | | Demonstration of the behaviour | HCS modelled by facilitator in all activities, & by trainees in various real/role play activities. |
|  | 7 | Associations | 7.1 | | Prompts & cues | Provided in the training room & as hand-outs. Also, attention drawn to language used by facilitator, i.e., modelling HCS. |
|  | 8 | Repetition & substitution | 8.1  8.3  8.6 | | Behavioural practice/rehearsal  Habit formation  Generalisation of a target behaviour | Prompt practice of all HCS in training room (artificial but safe environment).  Prompt practice of all HCS in real world (more challenging but real).  If have tried using skills with friend/relative, encourage to try out skills in workplace (e.g., with colleagues, patients/clients). |
| A1 Wider Determinants of Health & Exploratory Discussion | 6 | Comparison of behaviour | 6.2 | | Social comparison | 1^st^ opportunity to compare own practice & experiences with others. |
|  | 13 | Identity | 13.3 | | Incompatible beliefs | Draws attention to discrepancies between current/ past behaviour and view of self as health professional for example. So, when have challenges & frustrations around supporting someone, may lead to self-doubt regarding beliefs about competence. |
| A2.1 & 2.2 Recording & Listening back | 1 | Goals & planning | 1.2 | | Problem-solving | Listening to recordings elicits reflection on current practice, areas for improvement & strategies to develop skills. |
|  | 2 | Feedback & monitoring | 2.2  2.7 | | Feedback on behaviour  Feedback on outcome(s) of behaviour | Listening back provides opportunity for partner to provide evaluative feedback on performance.  Listening back is opportunity to discuss & receive feedback on what happened as a result of asking any ODQs as opposed to telling/suggesting or asking closed Qs. |
|  | 3 | Social support | 3.2  3.3 | | Social support (practical)  Social support (emotional) | Working in pairs to undertake & review recording task, elicits:  tips for practice & & sharing task (3.2);  someone listening & offering praise & encouragement (3.3). |
|  | 6 | Comparison of behaviour | 6.2 | | Social comparison | Listening back to self & partner allows comparison of both performances & outcomes related to these. |
|  | 13 | Identity | 13.2  13.3 | | Framing/reframing  Incompatible beliefs | Listening back following trainer demonstration of skills provides a new perspective on using skills hence changing cognitions related to using the skills.  Draws attention to discrepancies between current/ past behaviour and self-image as competent practitioner, in order to create discomfort. |
| A3 Beliefs Axis | 1 | Goals & planning | 1.2 | | Problem-solving | Encouraging analysis of factors influencing past/current behaviour in relation to beliefs, leads to shifts in understanding and intentions to change behaviour. |
|  | 13 | Identity | 13.2  13.3 | | Framing/reframing  Incompatible beliefs | Exploring own beliefs & listening to counterarguments provides new perspectives about current practice in order to change cognitions & emotions about changes to behaviour & skills required.  Draws attention to discrepancies between beliefs and past behaviour, in order to create discomfort & elicit new thinking. |
| A4 Response Styles | 1 | Goals & planning | 1.2 | Problem-solving | | Allows trainees to analyse own responses and factors influencing these, leading to generation of strategies to use new skills. |
|  | 4 | Shaping knowledge | 4.1 | Instruction on how to perform the behaviour | | Skills training supporting trainee to explore/agree on how to perform the behaviour, i.e., using ODQs. |
|  | 8 | Repetition & substitution | 8.1  8.2  8.4 | Behavioural practice/rehearsal  Behaviour substitution  Habit reversal | | Prompting use of ODQs in the training context in order to increase habit & skill.  Prompting substitution of the unwanted behaviour (suggesting/telling etc.) with ODQs.  Prompt rehearsal of ODQs to replace habitual use of suggestions/information-giving etc. |
|  | 9 | Comparison of outcomes | 9.3 | Comparative imagining of future outcomes | | Prompt comparison of possible outcomes following either suggesting/telling or asking ODQs, i.e., where does the conversation go? which is more effective for changing behaviour? |
|  | 13 | Identity | 13.2  13.3 | Framing/reframing  Incompatible beliefs | | Encouraging adoption of new perspective on using ODQs rather than other responses, in order to change cognitions about doing this – where it takes the conversation.  Responding to quotes highlights discrepancies between current behaviour (telling/suggesting etc.) and self-image as someone who explores someone’s world & wants to support change. |
| A5 Setting SMARTER Goals | 1 | Goals & planning | 1.1  1.2 | Goal setting (behaviour)  Problem-solving | | Working through example of setting/planning a behavioural goal.  Prompting exploration & analysis of factors influencing the behaviour & strategies to overcome barriers & facilitate action. |
|  | 8 | Repetition & substitution | 8.1 | Behavioural practice/rehearsal | | Prompting use of HCS, including ODQs & SMARTER goal setting within training context in order to increase habit & skill. |
|  | 13 | Identity | 13.2 | Framing/reframing | | Prompting adoption of a new perspective regarding making goals “SMARTER” & “owned” by individual in order to change cognitions about effectively supporting goal setting. |
| A6 SMARTER Planning for Change | 1 | Goals & planning | 1.1  1.2  1.4  1.8  1.9 | Goal setting (behaviour)  Problem-solving  Action-planning  Behavioural contract  Commitment | | Setting & agreeing behavioural goals in pairs.  In pairs, prompting each other to identify and analyse factors influencing behaviour & generating strategies to overcome barriers & facilitate change.  In pairs, prompting detailed (SMARTER) planning of behaviour.  Individual creates a written specification of the behaviour to be performed by completing the SMARTER planning for change sheet.  By asking individuals to state their goals, or their intention to reach their goal, it elicits a commitment to change their behaviour. |
|  | 2 | Feedback & monitoring | 2.2  2.3 | Feedback on behaviour  Self-monitoring | | By working in pairs, individuals can provide evaluative feedback to each other on performance of the behaviour (setting a SMARTER goal)  As part of SMARTER planning individuals can be supported to identify ways of monitoring changes made. |
|  | 3 | Social support | 3.2  3.3 | Social support (practical)  Social support (emotional) | | Working in pairs to support each other to set a SMARTER goal may include:  questions to encourage reflection on barriers/ solutions etc.  praise, encouragement, listening, empathy & sharing experiences. |
|  | 7 | Associations | 7.1 | Prompts/cues | | SMARTER planning for change sheet provides prompts/cues for setting goal(s). |
|  | 8 | Repetition & substitution | 8.1 | Behavioural practice/rehearsal | | Working in pairs provides each with the opportunity to practise asking ODQs, listening, supporting SMARTER planning & reflecting on usefulness of HCS. |
|  | 15 | Self-belief | 15.1  15.2  15.3  15.4 | Verbal persuasion about capability  Mental rehearsal of successful performance  Focus on past success  Self-talk | | Working in pairs provides opportunity for using a range of strategies to boost self-belief, including challenging self-doubts (15.1), imagining successful performance of behaviour (15.2), reviewing past successes (15.3), prompting positive self-talk before & during behaviour (15.4). |
|  | 16 | Covert learning | 16.2 | Imaginary reward | | Working in pairs provides opportunity to imagine performing the behaviour in real-life followed by a pleasant/desirable consequence (e.g., empowerment of patient following a HC; increased job satisfaction; healthier patient). |
| A7 Reviewing SMARTER Planning for Change sheets & Practising HCS | 1 | Goals & planning | 1.2  1.5  1.6  1.9 | Problem-solving  Review behaviour goal(s)  Discrepancy between current behaviour & goal  Commitment | | In pairs, prompting each other to identify and analyse factors influencing behaviour & generating strategies to overcome any barriers & facilitate further change.  In pairs jointly review goal(s) & consider modifications to strategies or goal(s).  In pairs jointly review discrepancies between current behaviour (including context, frequency, duration, intensity) & previously set goal(s) or action plans.  In pairs, can support each other to reaffirm commitment to continue/restart change. |
|  | 2 | Feedback & monitoring | 2.2  2.3 | Feedback on behaviour  Self-monitoring of behaviour | | In pairs jointly engage in reflective feedback on performance of the behaviour.  SMARTER planning for change sheet is one method of recording behaviour change strategies & plans |
|  | 3 | Social support | 3.2  3.3 | Social support (practical)  Social support (emotional) | | Working in pairs to support each other to review a SMARTER goal may include:  questions to encourage reflection on barriers/ solutions etc.  praise, encouragement, listening, empathy & sharing experiences. |
|  | 7 | Associations | 7.1 | Prompts/cues | | SMARTER planning for change sheet provides prompts/cues for reviewing/revising goal(s). |
|  | 8 | Repetition & substitution | 8.1  8.3  8.6  8.7 | Behavioural practice/rehearsal  Habit formation  Generalisation of target behaviour  Graded tasks | | Working in pairs provides each with the opportunity to practise asking ODQs, listening, supporting SMARTER planning & review of goal(s), reflecting on usefulness of HCS.  Working in pairs provides opportunity to prompt rehearsal of behaviour in the same context so the context elicits the behaviour (e.g., using HCS with every patient).  Working in pairs provides opportunity to prompt performance of wanted behaviour in different situations (e.g., used HCS with own family, now start to incorporate into professional practice).  In pairs can review how to set increasingly difficult, but realistic tasks, to work towards performance of the behaviour & achievement of goal(s). |
|  | 13 | Identity | 13.3 | Incompatible beliefs | | In pairs can review discrepancies between current behaviour & self-image in order to create discomfort (e.g., if not undertaken desired change, but sees themselves as a person who does the desired behaviour). |
|  | 15 | Self-belief | 15.1  15.2  15.3  15.4 | Verbal persuasion about capability  Mental rehearsal of successful performance  Focus on past success  Self-talk | | Working in pairs provides opportunity for using any of these strategies to boost self-belief, e.g., challenging self-doubts (15.1), imagining successful performance of behaviour (15.2), reviewing past successes (15.3), prompting positive self-talk before & during behaviour (15.4). |
|  | 16 | Covert learning | 16.2 | Imaginary reward | | Working in pairs provides opportunity to imagine performing the behaviour in real-life followed by a pleasant/desirable consequence (e.g., empowerment of patient following a HC; increased job satisfaction; healthier patient). |
| A8 Introduction to BCTs | 3 | Social support | 3.2  3.3 | Social support (practical)  Social support (emotional) | | Working in small groups to support each other to review use of BCTs may include:  Asking questions to encourage reflection on barriers to use / past experiences etc. (3.2);  listening, empathy & sharing experiences (3.3). |
|  | 9 | Comparison of outcomes | 9.1  9.3 | Credible source  Comparative imagining of future outcomes | | Provide list of BCTs identified in the literature as being generally effective in supporting change.  Prompt trainees to imagine & compare possible outcomes of using HCS to support patients to incorporate BCTs into change plans, i.e., more likely to make sustainable changes. |
|  | 15 | Self-belief | 15.3 | Focus on past success | | Working in small groups provides opportunity to reflect on previous successes in using BCTs to support behaviour change. |
| A9 SMARTER Team Challenge | 1 | Goals & planning | 1.1  1.2  1.4 | Goal setting (behaviour)  Problem-solving  Action-planning | | Opportunity to practise asking ODQs to support SMARTER goal setting.  Opportunity for everyone to analyse factors influencing behaviour and generate questions to support behaviour change.  Opportunity to prompt detailed planning of performance of the behaviour. |
|  | 2 | Feedback & monitoring | 2.2 | Feedback on behaviour | | Each team provides evaluative feedback on the performance of the other team, i.e., how successfully they supported SMARTER goal setting. |
|  | 3 | Social support | 3.3 | Social support (emotional) | | Taking a team approach to supporting SMARTER goal setting provides a safe, shared environment to practise HCS. |
|  | 8 | Repetition & substitution | 8.1 | Behavioural practice/rehearsal | | Opportunity to practise all 4 HCS. |
|  | 10 | Reward & threat | 10.4 | Social reward | | Facilitator (& other team) can congratulate both teams on effort & progress in performing required behaviours – using HCS to support SMARTER goal setting. |
| A10 Creating own Resource – combining HCS, philosophy & BCTs | 1 | Goals & planning | 1.2 | Problem-solving | | In small groups, reflecting on HCS training overall & how to most effectively graphically represent this to prompt future HCS use. |
|  | 3 | Social support | 3.2  3.3 | Social support (practical)  Social support (emotional) | | Working in small groups to support each other to design a resource includes use of:  questions to encourage reflection on training.  praise, encouragement, listening, empathy & sharing ideas. |
| Comparison of T1&T2 evaluation sheets | 1 | Goals & planning | 1.2  1.6 | Problem-solving  Discrepancy between current behaviour & goal | | By comparing pre- & post-training sheets, trainees are able to analyse factors influencing their responses and reflect on how to increase use of new skills.  Comparison of sheets draws attention to discrepancies between current practice and goals for implementing HCS in the future. |
|  | 2 | Feedback & monitoring | 2.3 | Self-monitoring of behaviour | | Comparing the self-completed sheets provides opportunity for recording own behaviour, and support their behaviour change strategy. |
|  | 8 | Repetition & substitution | 8.1 | Behavioural practice/rehearsal | | Completing & comparing the sheets prompts practice of the behaviour, e.g., forming ODQs. |
|  | 13 | Identity | 13.3 | Incompatible beliefs | | Comparing the sheets draws attention to discrepancy between past behaviour & self-image in order to create discomfort. |
| Reflection on training & next steps  Provision of laminated hand-outs | 1 | Goals & planning | 1.1  1.2  1.4  1.8 | Goal setting (behaviour)  Problem-solving  Action-planning  Commitment | | Trainees encouraged to agree goals for practising HCS.  Trainees prompted to review barriers & facilitators to incorporating HCS into practice (can include ‘relapse prevention’).  Trainees can be encouraged to make detailed plans for practising HCS, e.g., context, frequency.  Trainees asked to verbalise commitment to changing behaviour & using HCS. |
|  | 3 | Social support | 3.2  3.3 | Social support (practical)  Social support (emotional) | | Facilitator provides social support with:  questions to encourage reflection on barriers/ solutions etc.  praise, encouragement, listening, empathy & sharing tips. |
|  | 7 | Associations | 7.1 | Prompts/cues | | Laminated hand-outs act as social stimulus for prompting HCS use. Trainees encouraged to think about & discuss where they might keep/display them. |
|  | 8 | Repetition & substitution | 8.1  8.3  8.7 | Behavioural practice/rehearsal  Habit formation  Graded tasks | | Prompt trainees to practise HCS at every opportunity in order to increase habit & skill.  Prompt trainees to use HCS in their routine professional practice so that it becomes automatic.  Encourage trainees to set easy-to-perform tasks, making these increasingly difficult but achievable over time, e.g., build up to using HCS in all situations, even the most challenging. |
|  | 12 | Antecedents | 12.5 | Adding objects to the environment | | Displaying laminated hand-outs in the workplace facilitates performance of the behaviour, HCS use. |
|  | 13 | Identity | 13.1  13.2 | Identification of self as role model  Framing/reframing | | Opportunity to encourage trainee to reflect on role within wider team who might not have been trained.  Opportunity to reflect on adopting a new perspective on professional practice, in order to change cognition about role in supporting change & use of HCS. |
|  | 15 | Self-belief | 15.1 | Verbal persuasion | | Facilitator can empower trainee & raise self-efficacy. |
|  |  |  |  |  | |  |

**Appendix 2: Pre- and post- MECC HCS training evaluation forms**

**Pre- Training Evaluation**

| **Healthy Conversation Skills Evaluation**  **Please circle one number for each item**  **On a scale of 1 – 10 how confident do you feel about supporting individuals to make a lifestyle change?**  1 2 3 4 5 6 7 8 9 10  (Not confident) (Very confident)  **On a scale of 1 – 10 how important is it for you to support individuals to make a lifestyle change?**  1 2 3 4 5 6 7 8 9 10  (Not important) (Very important)  **On a scale of 1 – 10 how useful do you think the conversations you have are at supporting individuals to make a lifestyle change?**  1 2 3 4 5 6 7 8 9 10  (Not useful) (Very useful) |
| --- |

ID No:

***Please turn over***

Below are four things that patients might say in their physiotherapy appointments. Please write in the bubbles below the next thing you might say to support this individual to make a lifestyle change.

“I need to lose weight, but I don’t like vegetables.”

***You say:***

“I should cut down on my alcohol intake, but my

partner likes to open a bottle of wine after work.”

***You say:***

“I’ve lost count of the number of times

I’ve tried to stop smoking—it’s hopeless!”

***You say:***

“I just don’t seem to have time to do any exercise.”

***You say:***

**Post- Training Evaluation**

| **Healthy Conversation Skills Evaluation**  **Please circle one number for each item**  **On a scale of 1 – 10 how confident do you feel about using the skills you learnt on this course, in conversations with individuals to support them to make a lifestyle change?**  1 2 3 4 5 6 7 8 9 10  (Not confident) (Very confident)  **On a scale of 1 – 10 how important is it for you to support individuals to make a lifestyle change?**  1 2 3 4 5 6 7 8 9 10  (Not important) (Very important)  **On a scale of 1 – 10 how useful do you think the conversations you have are at supporting individuals to make a lifestyle change?**  1 2 3 4 5 6 7 8 9 10  (Not useful) (Very useful) |
| --- |

ID No:

Below are four things that patients might say in their physiotherapy appointments. Please write in the bubbles below the next thing you might say to support this individual to make a lifestyle change.

“I need to lose weight, but I don’t like vegetables.”

***You say:***

“I should cut down on my alcohol intake, but my

partner likes to open a bottle of wine after work.”

***You say:***

“I’ve lost count of the number of times

I’ve tried to stop smoking—it’s hopeless!”

***You say:***

“I just don’t seem to have time to do any exercise.”

***You say:***

| 1 | 2 | 3 | 4 | 5 |
| --- | --- | --- | --- | --- |
|  |  |  |  |  |

**How valuable did you find this training?**  (one being the lowest of value and five the highest):

**What could be done to improve this training?**

Thank you for your time to feedback.

**Appendix 3: Coding matrix for responses to health and wellbeing related statements by hypothetical patients**

| **1st Response – Code** | **Description** | **Example** |
| --- | --- | --- |
| 0. Unable to code | The individual has not responded appropriately—i.e., they haven’t written what they would say, or they have reworded the statement rather than given a response. Or no response. | e.g., “I’d probably say something about exercising with their children”. Or “Find out if they know about our courses and give them a leaflet”. |
| 1. Telling / suggestions (giving information)  Signposting | Telling someone what to do; telling them something about themselves; giving information, including specific suggestions about what someone could try, or offering options. Might start with ‘what/how about’, ‘what if’ or ‘why don’t you’. | e.g., “Get a recipe book.” Or “It’s never too late to learn.” Or “Tell me about your day”. Or “Try running up and down the stairs everyday”. Or “How about walking to work”. |
| 2. In my/others experience | A statement with a specific example of how the responder deals with a situation, including agreeing with the statement, OR how others might deal with it. Could be viewed as “normalising” the behaviour. **Not** if they demonstrate own knowledge (ie telling, Code as 1), but when share own or others’ behaviour. | e.g., “I try to build it into my day.” Or “I find it difficult too.” Or “This is what I usually do…” Or “Other people feel like that & this has worked for them.” |
| 3. Reflection /empathy  (See Code 7) | A statement that indicates an understanding of the person, or their situation. Can be repeating back what they’ve said in different words or clarifying understanding. (If precedes an ODQ, Code as 7). | e.g., “That must be difficult.” Or “Seems like you’d really like to do more exercise” |
| 4. Closed Question | A question with ‘yes’ or ‘no’ as possible answers | e.g., “Would you be interested in attending a workshop?” Or “Do you feel you have to go to a gym to exercise more?” |
| 5. Open Question (other) | A question that requires more than just a ‘yes’ or ‘no’ answer. May test knowledge but does not support people to explore their current behaviour, verbalise the benefits or barriers to change, or to come up with their own solutions. Often starts with ‘why’, ‘when’, ‘who’, which or ‘where’. Can start with What/How if not exploratory/empowering. | e.g., “Why can’t you fit that into your life?” Or “When are quiet times for your family?” Or “Where do you do your shopping?” |
| 6. Open Discovery Question (But **not** 1st response) | The individual has provided an Open Discovery Question (see Code 7) somewhere in their response, but it is **not** the first thing written down. **Except** if 1st response is empathy (Code as 7). **NOT** if ODQ is lost in list of unrelated suggestions (code as 1). | e.g., “Would you like to learn how to cook and what to eat? How would it benefit your family’s life?” |
| 7. Open Discovery Question | A question that requires more than just a ‘yes’ or ‘no’ answer. Supports people to explore their current behaviour, verbalise the benefits or barriers to change, come up with their own solutions or make their own plan, i.e., is empowering. Begins with **‘what’** or **‘how’**. **NOT** ‘how about/what about’ or ‘what if’ (this is telling, Code as 1). Include when directly following empathy. | e.g., “What could you do to change this?” Or “How do you think you might find out?” |

**Appendix 4: Six- to twelve- week follow-up call semi-structured interview script**

*Consent for the conversation to be recorded:*

*Consent given to record:* Yes / No

I’d like you to think back to the Making Every Contact Count Healthy Conversation Skills training and the skills you heard about. Remember we talked about Open Discovery Questions beginning with how and what, and how to incorporate these into conversations with individuals? We hope that you found the training useful and would like to know a bit more about how you are using the skills from the training. Since we saw you last hopefully you’ve had a chance to talk with your patients about their health behaviours or changing their behaviour in some way. What examples can you give about the conversations you’ve had? What is the story of how one went? Try to be as specific as possible.

**Tell the story…**

Questions to use if needed - remember you are assessing the trainee against the competencies rating rubric. Familiarise yourself with this rubric so questions you ask address these competencies in order to score them.

- How did the conversation start?
- What was the first thing you said? What else did you say? (ODQ)
- How long did it last?
- How did you feel it went?
- What worked well?
- Who did most of the talking in the conversation?
- How did you help this person plan for change?
- How did you help them set SMARTER goals?
- How useful did you find the training? What was of value or not of value?
- How do you think you have you used the skills you learnt on the training? What do you think you could do differently?
- What gets in the way of using your Healthy Conversation Skills? What is more important in your day-to-day work with clients?
- How have your conversations changed since attending the training?

We have no more questions. But I would like to thank you for answering these questions for us. We appreciate the time you have spent taking part in this evaluation.

**Six- to twelve- week follow up phone call competency coding tool**

TO BE COMPLETED INDEPENDENTLY BY 1 or 2 CODERS AFTER THE CONVERSATION. Write examples and/or quotes given by the physiotherapist in the spaces below. Rate success from 0-4 — refer to the Competencies Rating Rubric (see next page). Once completed, arrange to double-code and agree total score.

ID No:

| 1. **Asked Open Discovery Questions** | |
| --- | --- |
| E.g.    Rating: 0 1 2 3 4 | |
| 1. **Reflected on own practice** | |
| E.g.    Rating: 0 1 2 3 4 | |
| 1. **More time spent listening than giving information** | |
| E.g.    Rating: 0 1 2 3 4 | |
| 1. **Supported SMARTER goal setting** | |
| E.g.    Rating: 0 1 2 3 4 | |
| **Coding comments/ Quotes about training/HCS use:** | **Double coding**  **agreed total**  **(Out of 16):** |

**Competencies rating rubric for telephone interview (0 = worst to 4 = best)**

0 = No demonstration of Healthy Conversation Skills competency

1 = Some demonstration of Healthy Conversation Skills competency

2 = Moderate demonstration of Healthy Conversation Skills competency

3 = Good demonstration of Healthy Conversation Skills competency

4 = Strong demonstration of Healthy Conversation Skills competency

**1 Asked Open Discovery Questions**

**0** No evidence of using Open Discovery Questions at all in conversations

**1** Evidence of an awareness of the difference between Open Discovery Questions & other types of questions, though has not yet used Open Discovery Questions

**2 Limited** evidence of asking Open Discovery Questions, but **not** exploring context

**3 Some** evidence of asking Open Discovery Questions to explore context or plan change

**4 Substantial** evidence of asking Open Discovery Questions to explore context or plan change

**2 Reflected on own practice**

***(Reflection = describes own practice & provides a rationale for, or impact of, that practice)***

**0** No evidence of reflecting on own practice

**1** Evidence of reflecting on own practice, but doesn’t include changes since the training

**2 Limited** evidence of reflecting on own practice, e.g., can say what they do well since the training

**3 Some** evidence of reflecting on own practice, e.g., can say what they do well & not so well since the training

**4 Substantial** evidence of reflecting on own practice, e.g., can clearly articulate their strengths & identify areas for improvement in using the skills from the course

**3 More time spent listening than giving information**

***(When there’s a lack of explicit evidence for either of these, look at the bigger picture)***

**0** Evidence they spend the whole conversation giving information rather than listening

**1** Evidence they spend more time in conversations giving information than listening

**2** Evidence they spend equal amount of time in conversations giving information as listening

**3** Evidence they spend a little more time in conversations listening than giving information

**4** Evidence they spend substantially more time in conversations listening than giving information

**4 Supported SMARTER goal setting**

**0** No evidence of supporting planning

**1** No evidence of supporting planning, but evidence that they would like to

**2** Evidence of supporting planning by asking questions but not using the SMARTER technique **OR**

Evidence of using **SMARTER** techniques, but not encouraging people to make their own plans

**3** Evidence that they support **SMARTER** planning by asking questions to encourage people to come up with their own plans

**4** Evidence that they support **SMARTER** planning by asking questions to encourage people to come up with their own plans & has followed-up / intends to follow-up on those plans
